# Supplementary material for: Polyelectrolyte Coating of Ferumoxytol Differentially Impacts the Labeling of Inflammatory and Steady-State Dendritic Cell Subtypes
Source: Biomedicines. 2022 Dec 5;10(12):3137. doi: 10.3390/biomedicines10123137 (PMC9776020; doi:10.3390/biomedicines10123137)
Supplement: Supplementary file 1 [file biomedicines-10-03137-s001.zip › biomedicines-2057770-supplementary.pdf]

Supplemental Material

Polyelectrolyte Coating of Ferumoxytol Differentially Impacts the Labeling of Inflammatory and Steady-State Dendritic Cell Subtypes

Nehar Celikkin [1,2], John E. Wong [3], Martin Zenke [1,2], and Thomas Hieronymus [1,2,4*]

[1] Institute for Biomedical Engineering, Department of Cell Biology, RWTH Aachen University Hospital, Pauwelsstrasse 30, 52074 Aachen, Germany.
[2] Helmholtz Institute for Biomedical Engineering, RWTH Aachen University, Pauwelsstrasse 20, 52074 Aachen, Germany.
[3] Institute of Physical Chemistry, RWTH Aachen University, Landoltweg 2, 52056 Aachen, Germany
[4] Institute for Cell and Tumor Biology, RWTH Aachen University Hospital, Pauwelsstrasse 30, 52074 Aachen, Germany.

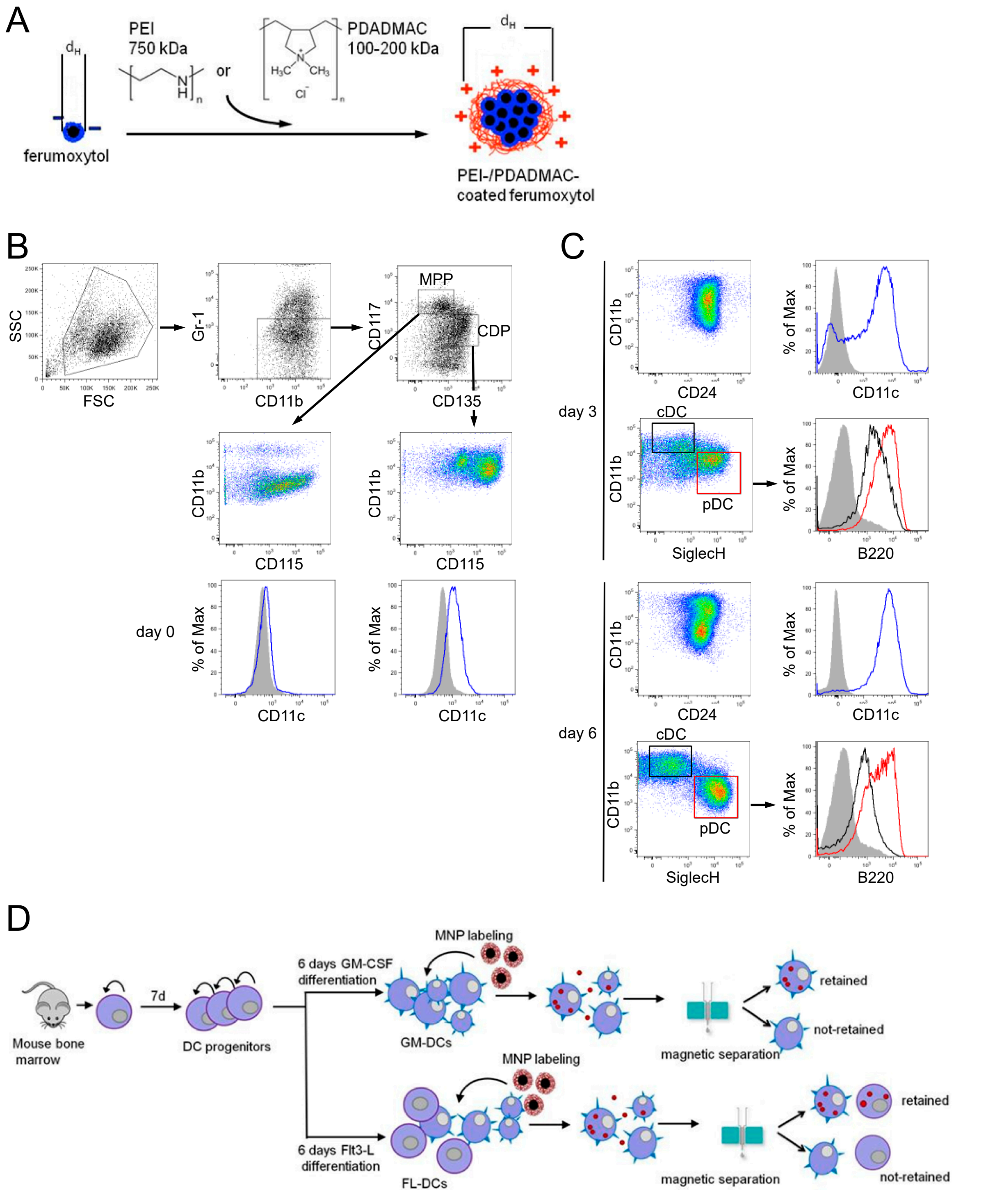

Figure S1. DC labeling with PE-coated and uncoated ferumoxytol. (A) Schematic representation of PE coating of ferumoxytol MNPs using PEI and PDADMAC; kDa, kilodalton; d_H, hydrodynamic diameter.

(B-D) The two-step culture systems for differentiation of steady-state DCs (FL-DCs) and inflammatory DCs (GM-DCs) from *in vitro* amplified Flt3+ DC progenitors and labeling with MNPs. (B) Phenotypic characterization of Flt+ DC progenitors for steady-state FL-DC differentiation towards cDCs and pDCs. Flt+ DC progenitors were examined by staining for Gr-1, CD11b, CD115, CD117/c-Kit, CD135/Flt3. According to CD117 and CD135 expression of Gr-1 negative cells, hematopoietic multipotent progenitor (MPP) and common DC-restricted progenitor (CDP) cells were identified (upper panel). Characteristic expression of CD11b, CD115 (middle panel), and CD11c (lower panel) in MPP and CDP are also shown. (C) FL-DC development towards cDCs and pDCs on days 3 and 6 of differentiation using Flt3L was examined by staining for CD11b, CD11c, CD24, SiglecH, and B220. According to CD11c, CD11b and SiglecH expression cDC and pDC populations were identified that display distinct pattern of expression for CD24 and B220 (pDCs, red lines and boxes; cDCs, black lines and boxes). Gray histograms show isotype control staining. Representative flow cytometry data from 3-5 independent experiments are shown. (D) Outline of MNP labeling and magnetic separation of cells developed in the two-step culture systems.
